# Influence of suit size and air permeability on performance in ski jumping. Part I: wind tunnel measurements

**DOI:** 10.3389/fspor.2025.1693699

**Published:** 2025-10-30

**Authors:** Mikko Virmavirta, Sören Müller, Mario Kürschner, Veronica Bessone, Piotr Krężałek, Ola Elfmark

**Affiliations:** ^1^ Faculty of Sport and Health Sciences, Biology of Physical Activity, University of Jyväskylä, Jyväskylä, Finland; ^2^ International Ski and Snowboard Federation, Oberhofen am Thunersee, Switzerland; ^3^ Department of Strength, Power and Technical Sports, Institute for Applied Training Science, Leipzig, Germany; ^4^ Department of Biomechanics, Institute of Biomedical Sciences, University of Physical Culture, Krakow, Poland; ^5^ Centre for Elite Sport Research, Department of Neuromedicine and Movement Science, Norwegian University of Science and Technology, Trondheim, Norway

**Keywords:** ski jumping, performance, suit size, field measurements, safety, fairness

## Abstract

In the first of a two-part study, the purpose was to investigate the influence of suit size and air permeability on ski jumping performance using wind tunnel measurements and numerical simulations. Results demonstrated that suit size greatly influenced aerodynamic performance, with drag increasing by ∼4% and lift by ∼5% for every 2 cm increase in tolerance. Changes in air permeability remained within the limits of measurement accuracy. Numerical simulations revealed an average difference of 5.8 m between suits of different sizes, corresponding to an increase of 2.8 m per cm of tolerance, based on a simulated reference jump of 130 m at Granaasen HS-138 m. The numerical simulations also highlighted that factors such as length of the reference jump, wind conditions, and altitude influenced performance difference. In summary, this investigation underscores the importance of suit size, as well as restricted importance of air permeability, on ski jumping performance, thereby enhancing the understanding of the role of equipment in the sport.

## 1 Introduction

Ski jumping is a popular, primarily competitive sport in which the equipment plays an important aerodynamic role. The velocity and posture of the jumper–ski system and suit design influence the aerodynamic resistance and lifting forces (1). Innovation and development of ski jumping equipment are highly constrained by the rules and regulations set by the International Ski and Snowboard Federation (FIS) (2). The importance of suit size becomes apparent by looking into the disqualification data from the World Cup (WC). During the last two seasons (2023–2025), 139 male ski jumpers were disqualified from WC competitions, and 93% of these cases were due to unregulated suits. This issue culminated in the suspension of five jumpers from competition due to suit manipulation suspicion after the World Championship 2025 in Trondheim (3). Suit size is understood to be the most important factor, as it greatly influences performance (4). The current rule states that a ski jumping suit can be 4 cm larger than the circumference of the ski jumper at any point (5). Due to the great benefit of larger suits (4), most disqualifications arise from infringing this rule. Another important suit parameter is air permeability of the suit, which also contributes to many disqualifications each season. According to FIS regulations, a ski jumping suit should have an air permeability greater than 40 L s $m^{-2}$, and the difference between the front and back parts should not be greater than 12 L s $m^{-2}$ at any point. It is worth mentioning that these regulations are not based on scientific findings. However, the FIS must balance the safety and fairness of the sport by strict equipment regulations, even in the absence of scientific background. Given the complexity of ski jumping, safety and fairness often compromise each other, and both should be taken into account (6–8). A body-fitted suit, like those in alpine skiing, could eliminate speculations and disqualifications, increasing fairness. However, obtaining the same jump length currently reached would require greater inrun speeds, which could compromise athlete safety. Hence, equipment serves as a means of balancing safety and fairness in ski jumping. The actual influence of suit size and air permeability on performance remains poorly understood, most likely due to challenges associated with obtaining reliable data.

Investigating a real-world problem, such as how a ski jumping suit affects performance, within comparable settings, and still being able to understand the underlying mechanisms, requires balancing internal and external validity (9, 10). Due to the nature of ski jumping, high external validity can only be achieved in field settings, where internal validity is compromised. However, internal validity is also needed to understand the underlying mechanisms in controlled settings. Wind tunnel measurements have a long history in ski jumping research and have been frequently used for different purposes, although in limited cases to investigate the jumping suit (4, 11–14). A mannequin model could then be used despite its limitation to static positions, as reproducing the repeatability of the flight posture with ski jumpers in a wind tunnel is challenging. The pioneering experiments with a mannequin model were performed by Straumann in 1927 (15), who continued his work by introducing an optimal aerodynamic flight posture, which is similar to that used in modern ski jumping (16). Some aerodynamic effects of the jumping suits were reported already by Meier (17), whose film analysis of the world-record 176-m jump in Oberstdorf 1976 showed a much slower landing velocity compared to the take-off velocity. This certainly would not have been possible without the “parachute effects” produced by the jumping suits of that era, which feature a completely air-impermeable back part. In the following years, the ski jumping suits with laminated foam have become one of the most tightly regulated sports equipment. In terms of research on the effect of ski jumping suits, Chowdhury et al. (11) investigated the aerodynamics of ski jumping suits using a life-sized mannequin in a wind tunnel. First, a +5-cm-tolerance suit was compared to a tight fitted suit, concluding that a tight-fitting suit could provide a performance advantage over the larger suit. Meile et al. investigated the influence of ski jumping suit size on a reduced-scale model in a wind tunnel and compared the results with numerical simulations (4). Here, a comparison was done between a tightly fitted alpine suit, a normal-sized, and a large ski jumping suit. The actual size difference was not reported. In contrast to the findings of Chowdhury et al., Meile et al. found that the large ski jumping suit on average increased drag by 3.5% and lift by 2.6%, underlying the common understanding of the aerodynamic benefits of a large suit (18, 19).

There is a public consensus across many sports where aerodynamic forces are of importance that the air permeability of apparel significantly influences performance (20). However, no scientific consensus supports this view, and studies suggest that air permeability has, at best, minimal influence on aerodynamic performance in tightly fitted garments (20, 21). Ski jumping suits differs from other sports apparel—they are not tight-fitting and are composed of five laminated layers with a thickness of 4–6 mm. Hence, the aforementioned research does not necessarily apply to ski jumping, and research examining the effect of air permeability in ski jumping is scarce. However, both Hasegawa et al. (22) and Kataoka et al. (23) investigated the matter and reached similar conclusions: fabrics with high air permeability could actually improve performance, contradicting the current consensus.

To enhance both safety and fairness of ski jumping, FIS initiated a project to investigate the effect of ski jumping suits on performance. For this reason, a project that combines high internal validity from wind tunnel measurements and external validity from field tests has been conducted and presented in two parts. Here, in Part I, the purpose was to investigate the influence of suit size and air permeability on ski jumping performance in a controlled setup. For this reason, measurements were performed on a life-sized mannequin wearing five suits differing in size and air permeability, with skis in an open-jet wind tunnel. The data from the wind tunnel test were then used in numerical simulations to assess the practical influence of the results. This study is followed by Part II, in which suits with the same characteristics as those used in Part I were tested in the field.

## 2 Materials and methods

### 2.1 Wind tunnel measurements

The experiment was carried out in an aero acoustic open-jet wind tunnel at the Audi Wind Tunnel Centre (Ingolstadt, Germany). The wind tunnel has a jet dimension of 11 $m^{2}$ (width: 3.94 m; height: 2.79 m), a contraction ratio of 5.5:1, and a test section length of 10 m. The wind tunnel uses a 2.6-MW fan and produces wind speeds up to 83 ${\mathrm{ms}}^{-1}$ with a turbulence intensity of <0.3%. A six-component force balance was used to measure the forces with an accuracy of ±0.5 N, and the air temperature was maintained at 19 ${}^{\circ }$C ± 0.2 ${}^{\circ }$C.

#### 2.1.1 Mannequin design

The investigations were carried out with a 1:1 scale mannequin (height 183 cm, body weight 74 kg) with joints (*n* = 19), reflecting anatomical conditions with respect to joint properties. Adjustments on the mannequin were based on a mathematical model—a matrix depicting the dependencies of individual joint settings. This made it possible to set realistic flight postures for the transition phase after take-off and the second half of the glide (24). The skis used corresponded to a practical model compliant with FIS regulations (25) and were mechanically fixed to the mannequin so that forces could be transmitted directly through a rigid connection. Angle settings for the body and skis are detailed in Section 2.1.3.

#### 2.1.2 Suit construction

**Figure 1:**
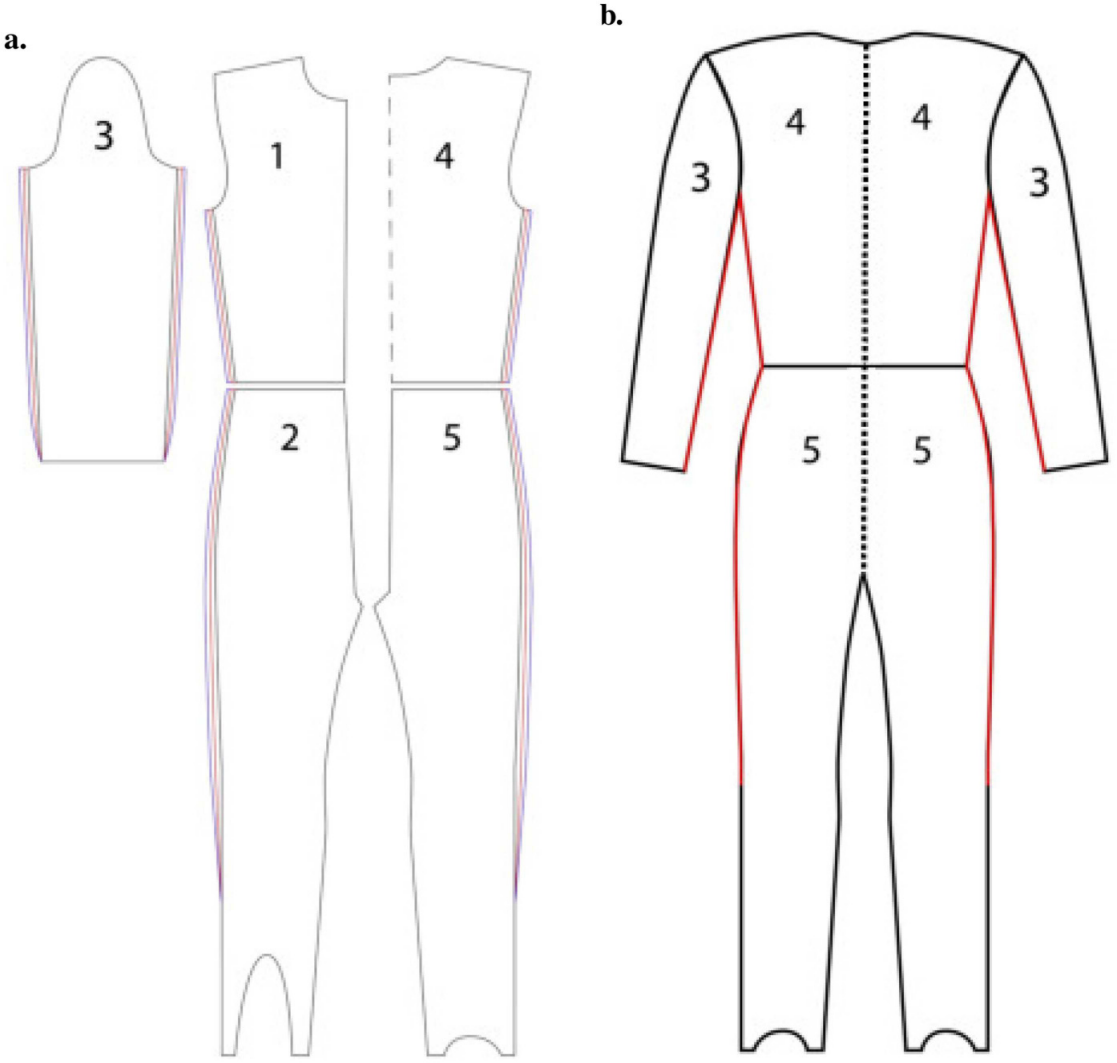
Graphical illustration of the suit construction. The five panels of a ski jumping suit are sewn together, with the suit adjustment locations indicated in **(a)**. Black and blue lines display how the suits were adjusted for the +2 cm and +6 cm suits. The back part of a fully constructed suit is shown in **(b)**, with red lines indicating adjusted areas and the dashed line representing the midline zipper used in the wind tunnel experiment. Panels 1 and 2 represent the front section of the suit, 3 corresponds to the sleeves, and 4 and 5 represent the back section.

The suits used, described in Section 2.1.3, were made from material provided by Meininger Jumpsuit (Bessenbach, Germany), and the same material was also used in Part II. A reference suit was set to be 4 cm larger than the circumference of the mannequin, according to the FIS equipment rules (25). The two other suits were adjusted by ±2 cm in the outer circumference relative to the reference suit, without varying the crotch length. The suit construction is shown in Figure 1.

**Figure 2:**
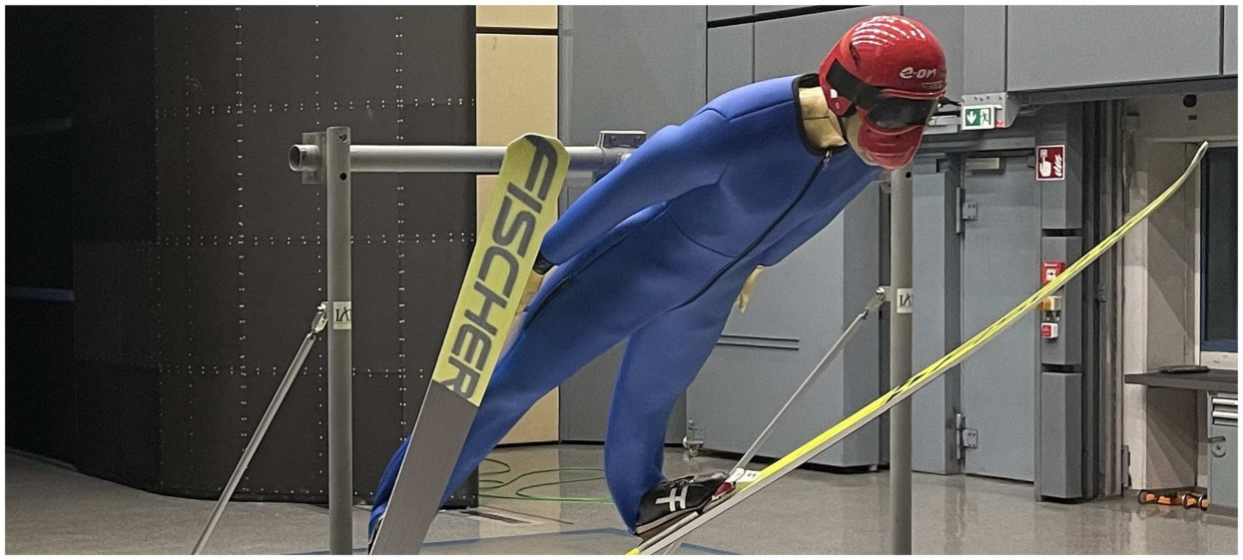
Picture of the mannequin mounted on the rack in the wind tunnel, visualizing the two modifications of the wind tunnel suits compared to FIS regulations: the black midline zipper and the hip zippers.

The size around the boots was not adjusted because the wind tunnel results were intended to be compared with field measurements in Part II, where coaches and suit makers deemed it unsafe to modify the suits in this area due to movement restrictions. Two adaptations were made to facilitate dressing the mannequin while mounted on the rack inside the wind tunnel. A midline circumferential zipper was added, allowing the suit to be changed quickly without having to remove the mannequin from the rack (Figure 1), and small zippers on both hips enabled adjustment of the hip angle if needed. These adaptations are visualized in Figure 2.

All suits followed the same construction procedure and were made by the same suit maker. Suits with air permeability of 20 L $s^{-1}\;m^{-2}$ and 80 L $s^{-1}\;m^{-2}$ followed similar construction by maintaining the same dimensions as the reference suit (+4 cm). A total of five suits with different sizes, in terms of tolerance and air permeability, were made for this investigation. Information about the suits is provided in Table 1.

**Table 1:** Information about the test suit size (tolerance) and air permeability.

Suit	Tolerance (cm)	Air permeability (L $s^{-1}$ $m^{-2}$)
1	+4	40
2	+2	40
3	+6	40
4	+4	20
5	+4	80

Consequently, Suits 1–3 were used to test changes in size while maintaining the same air permeability, while Suits 1, 4, and 5 were used to test changes in air permeability while maintaining the same size.

#### 2.1.3 Test protocol

**Figure 3:**
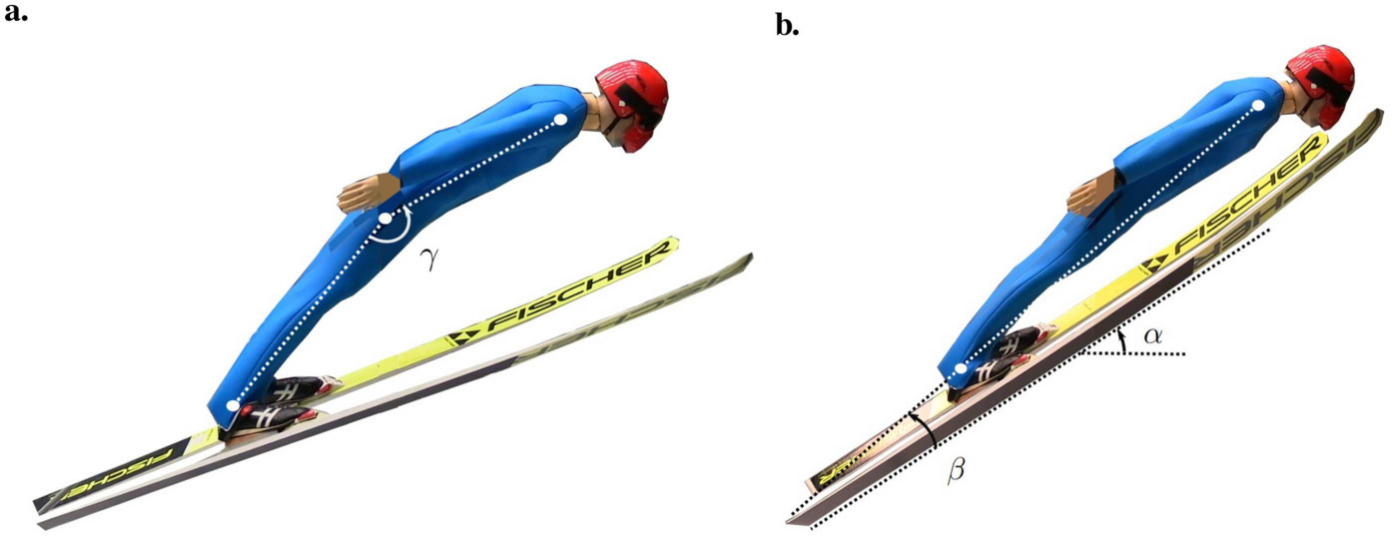
The two postures tested in the wind tunnel and angle definition describing the ski jumpers posture. **(a)** shows the posture of the transition (Pos 1) together with the hip angle γ and **(b)** the steady glide posture (Pos 2) together with the ski angle of attack α and the ski–body angle β.

To ensure that the suit tests were as relevant as possible, two different body–ski postures were defined: one from the transition phase (Pos 1) and the other from the steady glide phase (Pos 2). The angles used to determine the postures are visualized in Figure 3 and are described using common angle definitions from ski jumping wind tunnel studies (6, 26).

The postures examined were determined by kinematic analyses of the best 10 male ski jumpers (mean 1st–10th place) at the World Cups in Oberstdorf 2023 and 2024 and the Ski Flying World Cup in Oberstdorf 2023. A bent stance with a smaller α (Pos 1) was determined for the transition phase, and a stretched body posture with a larger ski angle of attack was selected for the steady glide phase (Pos 2). Three different angles of attack were investigated for each of the two postures by varying the mannequin and skis by a defined $5^{\circ }$ relative to the flow direction. The hip angle (γ) and the body–ski angle (β) remained unchanged, and α were set relative to the expected glide trajectory. The tests were carried out at three velocities corresponding to the expected velocity range in the two different postures. Information about the test setup is listed in Table 2.

**Table 2:** Information about the angle of attack (α), ski–body angle (β), hip angle (γ), and velocity used for the two postures in the wind tunnel experiment.

Ski–body posture	α (${}^{\circ }$)	β (${}^{\circ }$)	γ (${}^{\circ }$)	Velocity (m $s^{-1}$)
Pos 1	21.0 ∣ 26.0 ∣ 31.0	19.0	155.0	22.2 ∣ 25.0 ∣ 27.8
Pos 2	26.0 ∣ 31.0 ∣ 36.0	9.0	165.0	27.8 ∣ 30.6 ∣ 33.3

The velocity range used corresponded to a Reynolds number range between $1.4\times 10^{6}$ and $2.4\times 10^{6}$, with a set critical length of 1 m. Higher velocities were considered for Pos 2, as it can be achieved in competition. However, the pre-test showed that speeds over 33.3 m $s^{-1}$ (120 km $h^{-1}$) made the skis of the mannequin unstable, rendering the test setup unsafe. The three velocities were chosen for each configuration to investigate possible Reynolds number dependencies, which are important for comparisons with simulations and field tests (27). Blockage correction was not applied because the tests were performed in an open-jet wind tunnel. A picture was taken at every measurement from a stationary camera mounted on the side of the mannequin, outside the air flow, to assess body and ski postures. In both postures, Suit 1 was tested first and again at the end of the test series to check the repeatability of the test setup. The variation between the test and re-test was then used as the measurement uncertainty. This was only done for Suit 1 due to time restrictions. To obtain aerodynamic force values related exclusively to the mannequin with the suit, measurements of forces acting on the mounting frame without the mannequin were conducted. These measurements were performed for all tested velocities. The obtained drag and lift coefficient values for the frame were then subtracted from the corresponding total values for each measurement before further data analysis.

The air density (ρ) was calculated to 1.161 kg $m^{-2}$ based on the measured air temperature and the atmospheric pressure corresponding to the tunnel’s elevation in Ingolstadt (approximately 370 m a.s.l.). The aerodynamic coefficients $C_{d}A$ (drag area) and $C_{l}A$ (lift area) were determined from the measured drag and lift forces using the following relationship: $C_{d}A=2F_{d}/\rho v^{2}$ and $C_{l}A=2F_{l}/\rho v^{2}$, where $F_{d}$ and $F_{l}$ represent the drag and lift forces, respectively.

### 2.2 Numerical simulations

The computer simulation used in the present study was based on a complete ski jump model, with the ski jumper considered as a point mass (28). The simulation was a combination of first- and second-order integration methods for ski jump performance and employed a discrete time step of 0.02 s. It was carried out in four stages: (1) the inrun linear section and curve, (2) the linear take-off table section at the end of the inrun where the actual take-off takes place, (3) the transient flight phase (typically 0.25–0.50 s long period after take-off), and (4) the fully developed flight phase. The following parameters were used as input information: total mass and reference area of a ski jumper (based on the jumper’s body measurements), including skis, air density, coefficient of ski friction, and take-off force profile. Drag ($C_{d}A$) and lift ($C_{l}A$) area for the inrun position were obtained from previous studies (12, 13), and an aerodynamic polar function was created to establish $C_{d}A(t)$ and $C_{l}A(t)$ for the aerial phase using the results of the present study combined with the expected time development of the variables during the flight reported in previous studies (29, 30). The hill profile used in the simulation was the Granaasen (Trondheim, Norway) large hill with hill size (HS) 138 m (FIS certificate No. 289/NOR 39), reconstructed for the World Championships 2025 (31), as most jumps in the field test in Part II were to be conducted on this hill.

## 3 Results

### 3.1 Wind tunnel measurements

**Figure 4:**
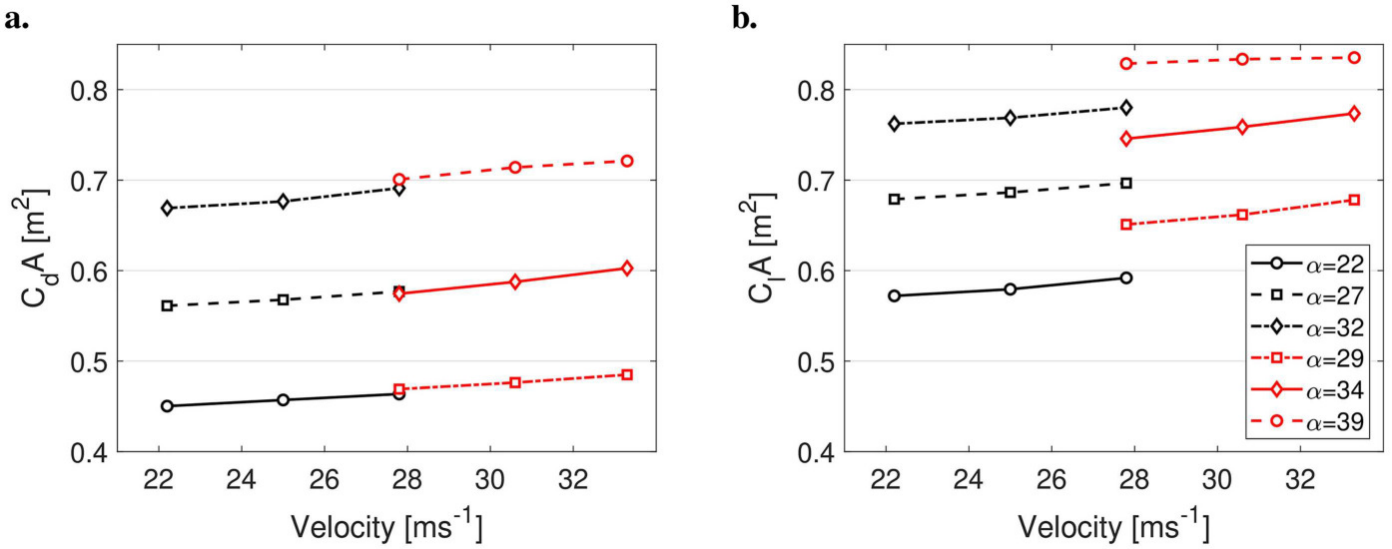
Drag area ($C_{d}A$) in **(a)** and lift area ($C_{l}A$) in **(b)** with respect to velocity for all ski angles of attack (α), set at the start of the test series. Posture 1 is highlighted in black, and posture 2 is highlighted in red.

Suit 1 (reference suit) was tested as the first and last suit for each posture, with the relative change in $C_{d}A$ and $C_{l}A$ in both postures presented in Supplementary Table S1. Altogether, the relative change between the test and the re-test was 0.81% ± 0.50%. This change was consistent across postures, velocities, and angles of attack. The relationships between velocity and both $C_{d}A$ and $C_{l}A$ were investigated to understand whether the data were Reynolds number (Re)-dependent or represented fully developed post-critical Re conditions to further understand how these data should be analyzed and used in numerical simulations. The average data from the first and second tests of Suit 1 are presented in Figure 4.

Both $C_{d}A$ and $C_{l}A$ exhibited a systematic increase of ∼1.5% for every 2.8 m $s^{-1}$. The increase was consistent for all suits (1.4% ± 0.3% on average). By investigating pictures from each test, the angle of attack (α) increased by $1^{\circ }$–$2^{\circ }$ for every velocity increase due to a higher pressure on the skis. As the changes were consistent across all test configurations, it was assumed that the tests were performed in a post-critical Re range. Hence, data on different velocities were averaged for further analysis.

#### 3.1.1 Suit differences

**Figure 5:**
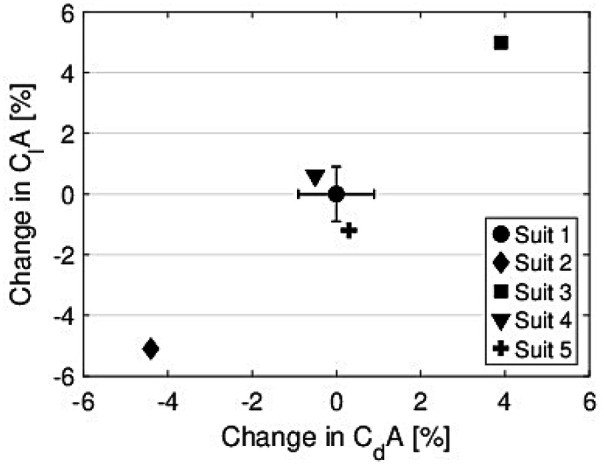
Percentage change in the drag area ($C_{d}A$) and lift area ($C_{l}A$) for all suits, relative to the reference suit (Suit 1). Data are averaged over all velocities, angles of attack (α), and both postures.

For the comparison of suits, the data were averaged over all angles of attack (α) and all velocities. The values of $C_{d}A$ and $C_{l}A$ for the five suits and two postures are presented in Supplementary Figure S1, and the average percentage changes between the suits are shown in Figure 5.

Due to a measurement error in the wind tunnel, the last two measurements in this series were not recorded. However, presenting the data in such manner allowed all measurements from Suit 2 to be used. Altogether, the differences between the suits were consistent across all α and velocities. Both $C_{d}A$ and $C_{l}A$ increased by a similar magnitude with suit size, while no noticeable (as no statistical significance tests were conducted) changes were observed for the air permeability test. The current data are also presented in Supplementary Table S2.

#### 3.1.2 Lift-to-drag ratio

**Figure 6:**
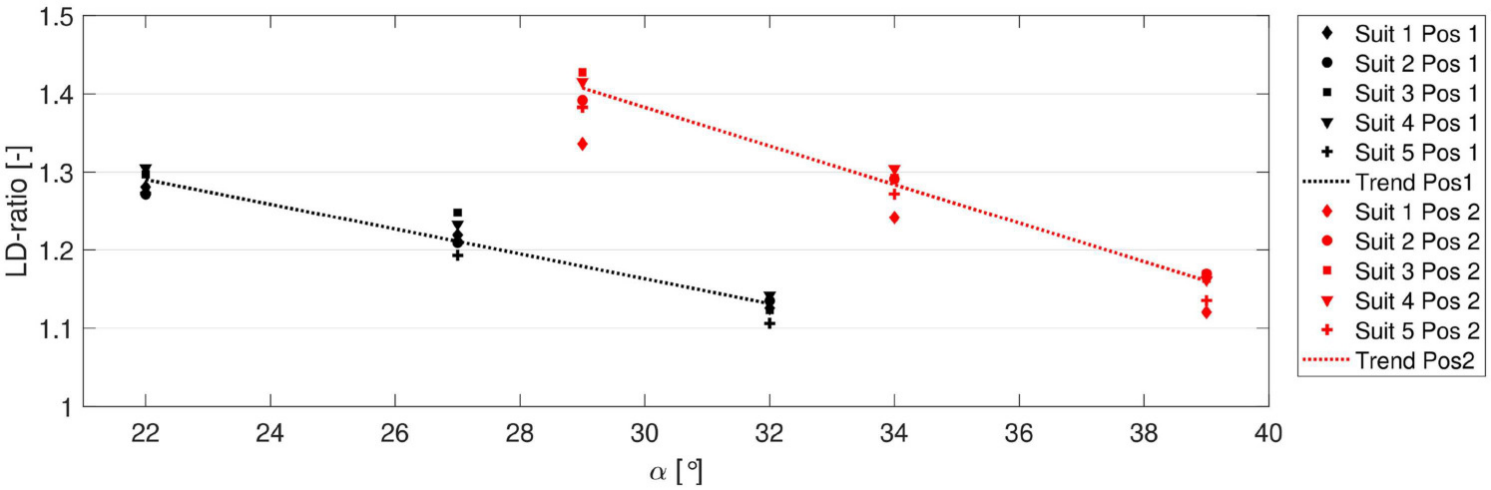
Relationship between the LD ratio and α. Pos 1 is shown in black, and Pos 2 is shown in red.

The relationship between the lift-to-drag ratio (LD ratio) and angle of attack (α) for all suits is shown in Figure 6.

All suits displayed similar relationships between the LD ratio and α for the two different postures. The relative difference in the LD ratio between Suit 1 and the rest of the suits was ≤1.7%. The LD ratio decreased by 0.02 per degree of α for both postures.

### 3.2 Numerical simulations

**Figure 7:**
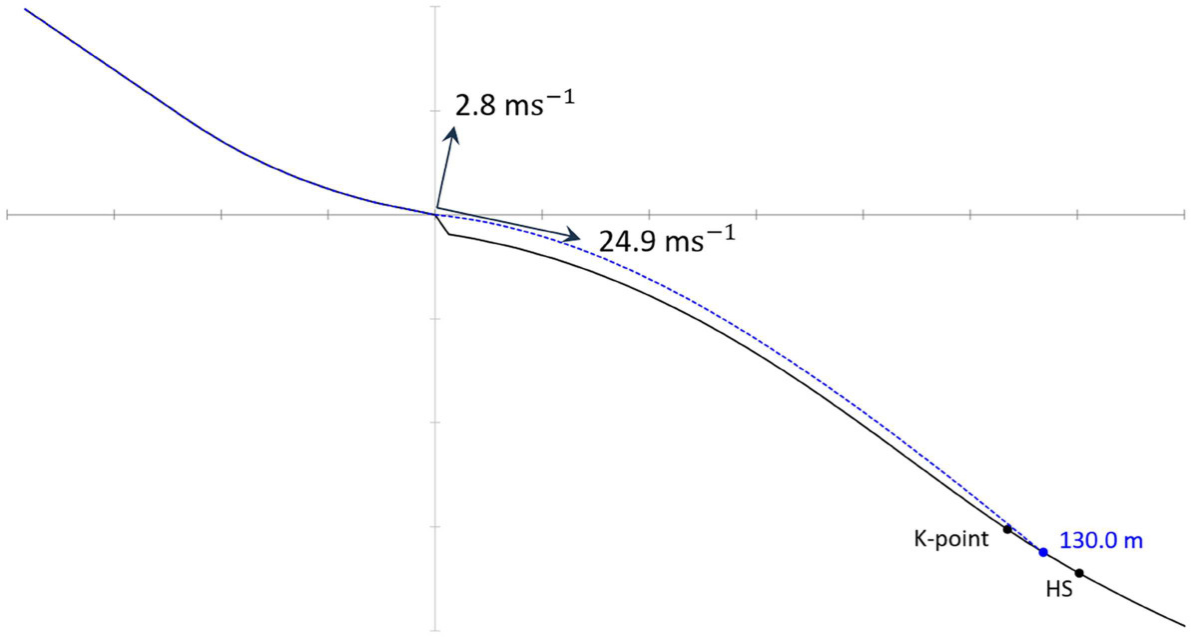
Visualization of the trajectory of the reference jump with Suit 1, displaying the simulated inrun speed and estimated take-off speed.

The reference jump for the simulations was established as a jump with Suit 1 with a length of 130 m, which denotes the length between the K-point and HS in the simulated hill. Information about unchanged parameters during all simulations is provided in Supplementary Table S3. A visualization of the simulated reference jump is shown in Figure 7.

**Figure 8:**
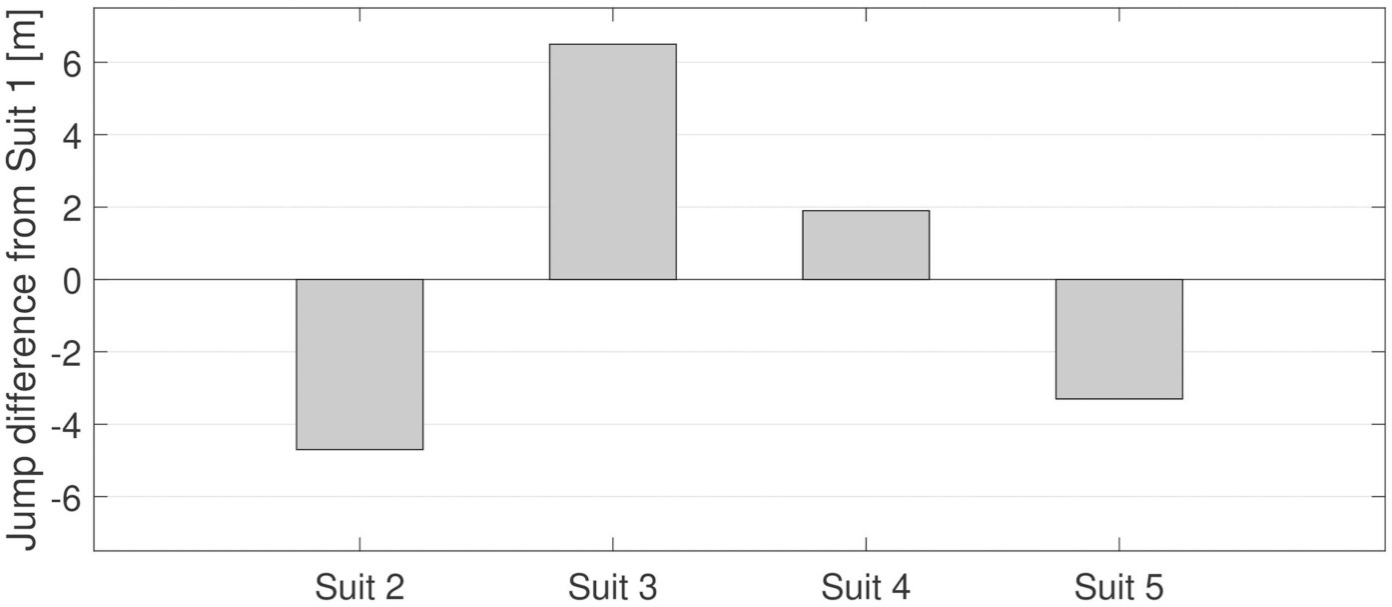
Simulated jump length difference from the reference jump at 130 m.

The aerodynamic polar function for the aerial phase was based on the wind tunnel measurements in combination with findings from earlier studies (29, 30) and is displayed in Supplementary Figure S2). After establishing the reference jump, all suits were simulated under the same conditions. The variation observed between the test to re-test of Suit 1 in the wind tunnel was ±0.8%. This difference accounted for a change in jump length of ±0.9 m when both lift and drag were changed similarly. Data for all simulations are given in Supplementary Table S4, and the jump length difference is shown in Figure 8.

The greatest differences were observed in the suits with variable sizes. The jump length decreased by 4.7 m for Suit 2 and increased by 6.5 m for Suit 3. Even if the wind tunnel measurements of air permeability suits (Suits 4 and 5) were within the measurement variation, changes of +1.9 and −3.3 m were estimated for Suits 4 and 5, respectively. Suit 4 benefited from a slight increase in lift area combined with a slight decrease in drag area. On the contrary, Suit 5 exhibited a slight decrease in lift area and an increase in drag area. Further analyses were only performed on the data from suits with different sizes (Suits 1–3) to reduce the number of simulations, facilitate result interpretation, and because the differences between the air permeability suits were considered small. In addition, only the suits with different sizes were include in the field tests in Part II.

**Figure 9:**
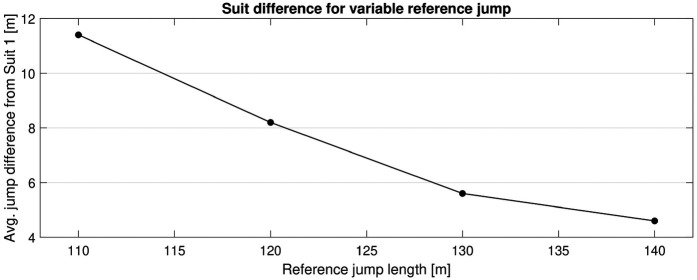
Average absolute jump length difference between the reference suit and suits ±2 cm in size for different reference jump lengths.

As mentioned previously, the reference jump was set to 130 m (between the K-point and HS) for this analysis to represent a normal jump distance in a competition. However, the length of the reference jump influences the results because the hill inclination varies. Figure 9 shows the average jump length difference between Suits 1 and 3 (Suit 1 vs. Suit 2 and Suit 1 vs. Suit 3) at varying reference jump lengths (110, 120, 130, and 140 m).

**Figure 10:**
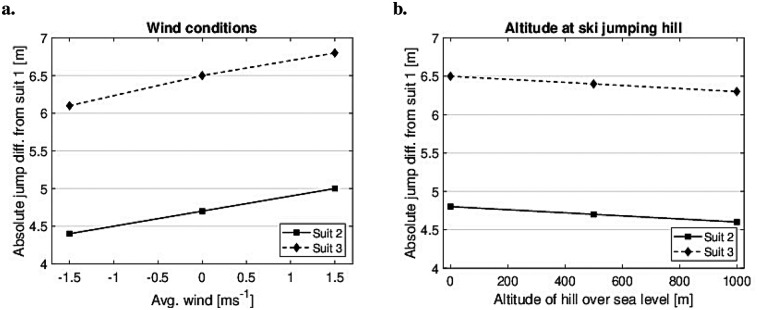
Absolute jump length differences due to external factors: wind conditions **(a)** and altitude **(b)**, for Suits 2 and 3 compared to Suit 1. The reference jump length for Suit 1 was set at 130 m.

Here, the reference jump is changed by changing the inrun length, i.e., the start gate. The difference in suit size decreases from 11.4 m with a reference jump of 110 to 4.6 m with a reference jump of 140 m. In addition, the influences of external factors, such as wind conditions and altitude, were investigated. Wind condition is an important performance factor (29, 32), and altitude changes the air density, again changing the flow regime, i.e., the aerodynamics. Figure 10 displays how suit size is influenced by these factors. In all cases, the reference jump is set to 130 m to obtain consistent results.

In terms of wind conditions, the largest differences were observed under headwind, where a 1 m $s^{-1}$ headwind increased the difference by ∼0.2 m. In terms of altitude, the largest differences were observed at sea level, decreasing by ∼0.1 m for every 500 m increase in altitude. Final simulations were performed in which all three suits were simulated to achieve a jump of 130 m by changing the inrun length (start gate) to investigate changes in key parameters. The data from these simulations are presented in Table 3 and Supplementary Figure S3.

**Table 3:** Suit comparison where the inrun speed is set to reach a jump of exactly 130 m by changing the inrun length, i.e., the start gate, with data on maximal height over ground ($h_{\max}$), perpendicular landing velocity ($V_{p}$), equivalent jump height ($h_{e}$), according to $V_{p}$, and the resultant landing velocity ($V_{l}$) for jumps to 130 m for the three suits.

Suit	Inrun speed	Inrun length	Start gate	$h_{\max}$	$V_{p}$	$h_{e}$	$V_{l}$
	(m $s^{-1}$)	(m)	(#)	(m)	(m $s^{-1}$)	(m)	(m $s^{-1}$)
1	24.91	87.20	14.00	3.65	4.22	0.91	29.25
2	25.13	88.45	16.50	3.65	4.34	0.96	29.83
3	24.55	85.25	10.10	3.65	4.03	0.83	28.80

The inrun length was here changed to precisely reach 130 m, and the estimated gate to which it corresponds is also presented. An increase of 0.22 m $s^{-1}$ in the inrun speed was needed for Suit 2 to reach 130 m, corresponding to a change of 2.5 gates. With Suit 3, one would need 0.36 m $s^{-1}$ less speed to reach 130 m, which corresponds to approximately four gates. The maximum height over ground ($h_{\max}$) was similar for all suits and is reach quite fast after take-off. Suit 2 exhibited an increase in perpendicular landing velocity ($V_{p}$) of 0.10 m $s^{-1}$, which corresponds to a equivalent jump height difference ($h_{e}$) of +0.05 m. Suit 2 also showed an increase in resultant landing velocity ($V_{l}$) of 0.58 m $s^{-1}$. Suit 3 exhibited a decrease in $V_{p}$ of 0.19 m $s^{-1}$, corresponding to a decrease in $h_{e}$ of 0.08 m, and a decrease in $V_{l}$ of 0.45 m $s^{-1}$.

## 4 Discussion

In the first of a two-part study, the purpose was to investigate the effect of ski jumping suits on performance. In this Part I, the influence of both suit size and air permeability was investigated. The main finding was that suit size largely influences aerodynamic performance, while air permeability exerted a limited effect on the results. Changing the suit circumference by +2 cm increased the lift area ($C_{l}A$) by ∼5% and the drag area ($C_{d}A$) by ∼4%. This was simulated to change the jump length by 5.8 m, which corresponds to an increase of 2.8 m/cm in suit tolerance, for a reference jump of 130 m. Changes in air permeability were in general small and more or less within the range of measurement variation seen from the test to the re-test of Suit 1. Suit 4 exhibited somewhat increased lift and less drag, while Suit 5 demonstrated somewhat lower lift and more drag. Due to this, Suit 4 was simulated to a 1.9-m-longer jump, while Suit 5 was simulated to a 3.3-m-shorter jump than the reference jump. Although the suits were made of similar fabrics, that of Suit 5 had a slightly different macrostructure, as the foam had larger holes to let more air pass through, which may have influenced the results of Suit 5 somewhat. In general, air permeability could have a small effect on overall performance, but these results indicate that it plays a minor role in ski jumping performance compared to suit size.

It is noteworthy that the wind tunnel measurement was done in a post-critical re-regime. Both lift and drag increased by ∼1.5% per 2.8 m $s^{-1}$, which was explained by an increase in α of $1^{\circ }$–$2^{\circ }$ due to a higher pressure on the skis for higher velocities. Due to this fact, data for all suits were averaged over all velocities in the analyses and comparisons. Moreover, velocity dependencies were not needed to be taken into account in the numerical simulations either. The average measurements of $C_{l}A$ and $C_{d}A$ from the wind tunnel of the reference suit were 0.72 and $0.59\;m^{2}$, respectively. For all suits, $C_{l}A$ values were in the region 0.55–0.89 $m^{2}$ and $C_{d}A$ values were in the region 0.43–0.79 $m^{2}$. This coincides well with previous wind tunnel and computational fluid dynamics (CFD) simulations (6, 26, 33) and also estimations done in the field by Elfmark et al. (34). Similarly, the absolute values of the LD ratio, from 1.1 to 1.5, were in line with the literature (4, 6, 26, 33, 34), and the relationship between the LD ratio and α was consistent. On average, the LD ratio was found to decrease by 0.02 per degree of increase in α, a trend similar to that found by Meile et al. for a similar body–ski posture (4).

Published investigations into the effect of suit size rarely are rare. This, combined with the fact that both suit materials and FIS regulations evolve over time, makes it difficult to directly compare results on the observed suit size effects. However, Meile et al. did compare a normal suit (within the current FIS regulations) to one they reported as a “suit with extreme width,” although they provide no further specification, in 2006. Drag increased on average by 3.5% and lift by 2.6% (4). The data from the current study showed that increasing the suit size by 2 cm resulted in a 4%–5% increase in drag and lift. Hence, it seems like one would gain even more on a larger suit with the current suit material and FIS regulations, compared to that reported in 2006. However, this remains a speculation because Meile et al. did not report the actual suit size difference in their study. Another important finding, not observed by Meile et al. (4), was that lift increased more than drag. In general, the aerial performance of a ski jump is defined by two factors: minimizing vertical velocity and maximizing horizontal velocity (34, 35). Vertical velocity is minimized by increasing the aerodynamic forces, and horizontal velocity is maximized by increasing the LD ratio. Hence, a larger suit benefits both. It mostly helps decrease vertical velocity, but since lift increases more than drag, it also increases the horizontal velocity.

The wind tunnel data were subsequently used in numerical simulations to understand the practical influence of the results and to provide comparative data for the upcoming study (Part II). A reference jump of 130 m, which is a normal competition length, falling between the K-point and HS, was chosen. However, it is clear that the length of this reference jump influences the result because the hill inclination changes, with the biggest differences observed for shorter jumps. This itself is an important finding, as it implies that the effect of equipment changes will be more significant in a competition where average jump length is shorter. As an example, during the 2025 Large hill World Championship competition held on the ski jumping hills in Granaasen (Trondheim) (36, 37), the average jump lengths for the top 10 men and women were 133.9 and 125.7 m, respectively. Based on the current simulations, increasing the suit size by +2 cm would increase the jump length by ∼5 m for men and ∼7 m for women. Moreover, the study explored external factors such as wind and altitude, discovering only minor variations. However, extreme combined values of both wind and altitude could still be influential (29). As an example, the difference at 1,000 m altitude with −2 m $s^{-1}$ wind, compared to a competition held at sea level with +2 m $s^{-1}$ wind (both reasonable examples), would change the expected suit difference by ∼1 m. This suggests that the fairest competitions are those with high altitude, tailwind, and long average jumps. In contrast, these types of competitions could compromise the safety because they require higher velocities. This highlights, once more, the compromise between safety and fairness in ski jumping.

Although the results of this investigation emphasize the consensus that suit size largely influences performance, its influence on safety is less clear. To explore this, suits of different size were simulated to 130 m by changing the inrun length. The highest speeds were reached with the smallest suit. This is because its aerodynamic performance was of lower quality, requiring more speed to reach a similar length. However, the maximum height over the ground was similar for all suits. Only minor differences in trajectories were observed in the last part of the glide, where the largest suit had the lowest trajectory. The difference in perpendicular landing velocity was roughly half the observed inrun velocity, which corresponded to an average difference in equivalent jump height of 0.07 m. Hence, the landing impact do not differ much between the suits for a jump of similar length. However, the landing impact is greatly influenced by the inclination of the landing area and the outrun radius (38). As the resultant landing velocity required to reach 130 m increases by ∼0.5 m $s^{-1}$ per increment in suit size, the centrifugal force in the landing will increase. A larger suit compromises fairness is due to the difficulty FIS has in controlling its dimensions. However, comparing the suits with regard to safety and landing impact, one could still argue that a larger suit is safer. In particular, when an athlete widens the angle between skis and landing area and executes a backward rotation during landing preparation, which will be easier with a larger suit, the resulting braking action softens the landing (39). This scenario requires less speed, leads to a lower trajectory during the second part of the glide, and results in a similar landing impact but lower impact after the landing due to lower centrifugal force in the outrun. However, this safety issue is more of a hill construction problem, as a steeper landing would solve this issue.

A separate finding also emerged from the suit size simulation in which all suits were simulated to achieve a jump length of 130 m. With Suit 3, one would need to start roughly four gates (or exactly 1.95 m) lower to reach 130 m, which is the only changed parameter in this comparison. The gate compensation in Granaasen is 7.45 points/m, giving a point difference between the suits of 14.5 points. However, the simulated difference between the suits when all parameters were kept constant (Figure 8) was 6.5 m, which corresponds to a point difference of 11.7 points. This indicates that the gate might be slightly over-compensated, with approximately 0.7 points per gate, which in many cases could influence the outcome of a competition.

This study has several notable limitations that should be considered when interpreting the results. A simplified experimental design was used, investigating the effects of suit size and air permeability independently. Suits combining different sizes with different permeabilities were not tested, preventing assessment of potential interactions between these parameters. Theoretically, the influence of permeability may be greater for larger suits due to the larger material surface area and volume of the space between the suit and the athlete’s body. Furthermore, the use of a static mannequin does not capture the subtle body position adjustments made by jumpers during actual flight. Only two body positions were studied, which do not represent the full range of configurations adopted during flight, especially in the transitional phase. In addition, wind tunnel testing does not replicate variable wind conditions present during actual competitions, which may modify the relative impact of suit parameters on aerodynamic performance. Testing was also limited to a single material type with different permeabilities and a small number of size variants, whereas real suits differ in seam construction and other features that affect aerodynamic behavior. Finally, the simulation model used simplified assumptions, such as ignoring crosswind and turbulence effects and treating the jumper as a point mass. Despite these limitations, the obtained results provide valuable insights into the influence of basic suit parameters on aerodynamic performance and constitute a solid foundation for further, more detailed research.

## 5 Summary

Altogether, this investigation demonstrates the importance of suit size and the restricted influence of air permeability on ski jumping performance. Enlarging the suit by 2 cm increased the lift and drag force by 5% and 4%, respectively. An increase in both aerodynamic forces and the LD ratio was seen, which provides a benefit by reducing vertical velocity and increasing horizontal velocity. The numerical simulations showed that this aerodynamic effect corresponded to an average jump length difference of 5.6 m, or 2.8 m/cm of increased tolerance. Air permeability had a limited effect on performance. Wind tunnel measurements showed differences within the expected measurement variations. However, these changes still resulted in jump differences of +1.9 for Suit 4 and −3.3 m for Suit 5. While air permeability may have a small effect on performance, its influence is significantly overshadowed by the effect of suit size. Numerical simulations confirmed that the equipment difference is the largest for shorter jumps, where the landing slope is steeper. A somewhat larger difference between the suits was also observed in headwind compared to tailwind, and at sea level compared to higher altitude. These findings suggest that the fairest ski jumping competitions would be those held at high altitude, with tailwind, and long average jumps. However, this condition could compromise the safety of the competition. Comparisons of jumps of similar jump lengths displayed only small differences in landing impact. Nevertheless, the fact that higher speed is needed to reach similar length with a smaller suit could still influence safety during the glide and outrun phases, and the real impact could be confirmed with additional testing of smaller suits in a controlled and safe setup.

## Data Availability

The original contributions presented in the study are included in the article/Supplementary Material, further inquiries can be directed to the corresponding author.

## References

[1] Elfmark O Ettema G Groos D Ihlen EA Velta R Haugen P, et al. Performance analysis in ski jumping with a differential global navigation satellite system and video-based pose estimation. Sensors. (2021) 21:5318. 10.3390/s21165318 34450758 PMC8399095

[2] Brownlie L. Aerodynamic drag reduction in winter sports: the quest for “free speed”. Proc Inst Mech Eng Part P. (2021) 235:365–404. 10.1177/1754337120921091

[3] NBC-Sports. Data from: World championships Trondheim: five Norway ski jumpers, three officials suspended (2025). Available online at: https://www.nbcsports.com/olympics/news/norway-ski-jumpers-suspended-suits-marius-lindvik-johann-forfang (accessed April 29, 2025).

[4] Meile W Reisenberger E Mayer M Schmölzer B Müller W Brenn G. Aerodynamics of ski jumping: experiments and CFD simulations. Exp Fluids. (2006) 41:949–64. 10.1007/s00348-006-0213-y

[5] FIS. Data from: Guidelines for measuring and control procedure (2024). Available online at: https://assets.fis-ski.com/f/252177/x/63d3b88394/guidelines-for-measuring-and-control-procedure-2024_25.pdf (accessed February 27, 2025).

[6] Müller W Platzer D Schmölzer B. Dynamics of human flight on skis: improvements in safety and fairness in ski jumping. J Biomech. (1996) 29:1061–8. 10.1016/0021-9290(95)00169-7 8817373

[7] Müller W. Determinants of ski-jump performance and implications for health, safety and fairness. Sports Med. (2009) 39:85–106. 19203132 10.2165/00007256-200939020-00001

[8] Elfmark O Sandbakk Ø Brevig M Ettema G. Performance and jump-to-jump development in the first female ski flying competition in history. Front Sports Act Living. (2024) 6:1366042. 10.3389/fspor.2024.1366042 38752211 PMC11094544

[9] Atkinson G Nevill AM. Selected issues in the design and analysis of sport performance research. J Sports Sci. (2001) 19:811–27. 10.1080/026404101317015447 11561675

[10] Schwameder H. Concepts in ski jumping biomechanics and potential transfer to other sports. In: *ISBS-Conference Proceedings Archive*. (2014).

[11] Chowdhury H Alam F Mainwaring D. Aerodynamic study of ski jumping suits. Procedia Eng. (2011) 13:376–81. 10.1016/j.proeng.2011.05.101

[12] Elfmark O Ettema G. Aerodynamic investigation of the inrun position in ski jumping. Sports Biomech. (2024) 23:455–69. 10.1080/14763141.2020.1871503 33533308

[13] Virmavirta M Kivekäs J Komi P. Ski jumping takeoff in a wind tunnel with skis. J Appl Biomech. (2011) 27:375–9. 10.1123/jab.27.4.375 21896946

[14] Virmavirta M Kivekäs J. Aerodynamics of an isolated ski jumping ski. Sports Eng. (2019) 22:1–6. 10.1007/s12283-019-0298-1

[15] Straumann R. Vom skiweitsprung und seiner mechanik. In: *Jahrbuch des Schweizerischen Ski Verbandes*. Bern, Switzerland: Selbstverlag des SSV (1927). p. 34–64.

[16] Straumann R. Vom skisprung zum skiflieg. Sport Zürich. (1955) 63:7–8.

[17] Meier R. Skifliegen-Schanzenbau. Dipl Arbeit. Institut für Biomechanik, ETH Zürich (1977).

[18] Schwameder H. Biomechanics research in ski jumping, 1991–2006. Sports Biomech. (2008) 7:114–36. 10.1080/14763140701687560 18341140

[19] Virmavirta M. Aerodynamics of ski jumping. In: Springer, editors. *The Engineering Approach to Winter Sports*. New York: Springer (2016). p. 153–81.

[20] Bardal LM Reid R. The effect of textile air permeability on the drag of high-speed winter sports apparel. Sports Eng. (2014) 17:83–8. 10.1007/s12283-013-0134-y

[21] Oggiano L Roar SL Morten BL Brian H. Air permeability and drag crisis on high tech fabrics for cross country ski competitions. Procedia Eng. (2012) 34:15–9. 10.1016/j.proeng.2012.04.004

[22] Hasegawa H Kawabata Y Murakami M Seo K Obayashi S. Effect of air permeability on the aerodynamic characteristics of ski jumping suits. Adv Exp Mech. (2018) 3:118–22. 10.11395/aem.3.0_118

[23] Kataoka Y Hasegawa H Murakami M Seo K Obayashi S. Flow behavior caused by air permeability of ski jumping suit fabric. In: *Proceedings*. MDPI (2020). Vol. 49. p. 109.

[24] Kreibich S. Präzisierung der Technikorientierung für die V-Skihaltung im Skispringen auf der Basis von Windkanaluntersuchungen. Leizig: Meyer & Meyer (2018). Vol. 12.

[25] FIS. Data from: Specifications for competition equipment (2023). Available online at: https://assets.fis-ski.com/f/252177/a8c13eeceb/specifications-for-cc_jp_nc_competiton-equipment_2023_24_marked-whole.pdf (accessed January 16, 2025).

[26] Schmölzer B Müller W. Individual flight styles in ski jumping: results obtained during Olympic games competitions. J Biomech. (2005) 38:1055–65. 10.1016/j.jbiomech.2004.05.038 15797587

[27] Elfmark O Reid R Bardal LM. Blockage correction and Reynolds number dependency of an alpine skier: a comparison between two closed-section wind tunnels. Proceedings. (2020). 49:19. 10.3390/proceedings2020049019

[28] Virmavirta M Kivekäs J. Effective use of a wind tunnel for ski jumping suit research. In: *Proceedings of the XXIIth ISB Congress*. South Africa: ISB Cape Town (2009).

[29] Virmavirta M Kivekäs J. The effect of wind on jumping distance in ski jumping–fairness assessed. Sports Biomech. (2012) 11:358–69. 10.1080/14763141.2011.637119 23072046

[30] Schmölzer B Müller W. The importance of being light: aerodynamic forces and weight in ski jumping. J Biomech. (2002) 35:1059–69. 10.1016/S0021-9290(02)00066-0 12126665

[31] FIS. Data from: Granaasen hill certificate (2025). Available online at: https://www.skisprungschanzen.com/photos/nor/trondheim_granaasen/HS138_2024.pdf (accessed March 25, 2025).

[32] Jung A Müller W Virmavirta M. A heuristic model-based approach for compensating wind effects in ski jumping. J Biomech. (2021) 125:110585. 10.1016/j.jbiomech.2021.110585 34233216

[33] Gardan N Schneider A Polidori G Trenchard H Seigneur JM Beaumont F, et al. Numerical investigation of the early flight phase in ski-jumping. J Biomech. (2017) 59:29–34. 10.1016/j.jbiomech.2017.05.013 28558914

[34] Elfmark O Ettema G Gilgien M. Assessment of the steady glide phase in ski jumping. J Biomech. (2022) 139:111139. 10.1016/j.jbiomech.2022.111139 35609493

[35] Jølstad PAH Gilgien M Elfmark O. Investigation of individual strategies in the aerial phase in ski jumping. Sci Rep. (2023) 13:22505. 10.1038/s41598-023-49683-0 38110490 PMC10728078

[36] FIS. Data from: Result world championships Trondheim—mens large hill individual competition (2025). Available online at: https://medias2.fis-ski.com/pdf/2025/JP/3224/2025JP3224RL.pdf (accessed April 01, 2025).

[37] FIS. Data from: Result world championships Trondheim—womens large hill individual competition (2025). Available online at: https://medias2.fis-ski.com/pdf/2025/JP/3223/2025JP3223RL.pdf (accessed April 01, 2025).

[38] Bessone V Petrat J Schwirtz A. Ground reaction forces and kinematics of ski jump landing using wearable sensors. Sensors. (2019) 19:2011. 10.3390/s19092011 31035683 PMC6539877

[39] Bessone V Schwirtz A. Landing in ski jumping: a review about its biomechanics and the connected injuries. J Sci Sport Exerc. (2021) 3:238–48. 10.1007/s42978-020-00096-9

