# Supplementary material for: Influence of suit size and air permeability on performance in ski jumping. Part I: wind tunnel measurements
Source: Front Sports Act Living. 2025 Oct 30;7:1693699. doi: 10.3389/fspor.2025.1693699 (PMC12611860; doi:10.3389/fspor.2025.1693699)
Supplement: Supplementary file 1 [file Datasheet1.pdf]

Supplementary Material

1 ADDITIONAL WIND TUNNEL RESULTS

Table S1 display the relative change in $C_l A$ and $C_d A$ in both postures between the first test and re-test of Suit 1.

Table S1. Percentage relative change (mean±SD) in lift area ($C_l A$) and drag area ($C_d A$) between the first test and re-test of the reference suit (Suit 1), averaged over all velocities and angle of attacks (α).

Posture	$C_l A$ change [%]	$C_d A$ change [%]	Total change [%]
1	0.84±0.59	0.85±0.77	0.84±0.61
2	0.62±0.29	0.92±0.41	0.77±0.34
Both	0.75±0.45	0.88±0.59	0.81±0.50

Average percentage change from Suit 1 is shown in Table S2.

Table S2. Average percentage change from Suit 1 in lift ($C_l A$) and drag ($C_d A$) area from the wind tunnel measurements.

	$C_l A$ change [%]	$C_d A$ change [%]
Suit 1±variation	0.0±0.8	0.0±0.8
Suit 2	-5.1	-4.4
Suit 3	5.0	3.9
Suit 4	0.6	-0.5
Suit 5	-1.2	0.3

The average values over all angles of attack (α) and all velocities of $C_d A$ and $C_l A$ for the five suits and two postures are presented in Figure S1, with exception of Suit 2 in Pos 2.

a. **b.**

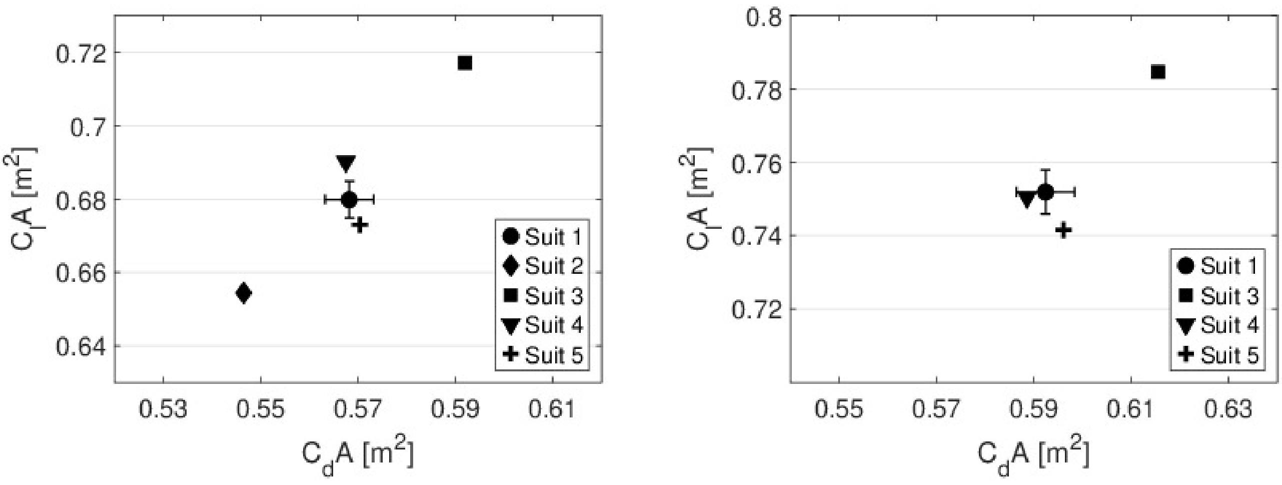

Figure S1. Drag area ($C_d A$) and lift area ($C_l A$) for the five different suits and both postures. Data averaged over three ski angles of attacks (α) and three velocities. Data from Pos 1 is shown in **a** and Pos 2 in **b**. Error bars indicating measurement uncertainty.

Due to a measurement error in the wind tunnel, the last two measurements in this series were not recorded. Hence, calculating an average over all velocities was not possible.

2 ADDITIONAL INFORMATION ON NUMERICAL SIMULATIONS

Table S3 shows values used for parameters that remained unchanged for all simulations.

Table S3. Values on parameters on ski jumper, inrun and air temperature that remained unchanged during all simulations in this investigations.

Parameter	Value
Ski jumpers body mass	$60.0\,\mathrm{kg}$
Ski jumpers height	$1.73\,\mathrm{m}$
Ski jumpers BMI	$20.05\,\mathrm{kg\,m^{-2}}$
Ski length	$2.44\,\mathrm{m}$
Inrun friction coefficient (μ)	0.035
Inrun drag area (C_dA_{Inrun})	$0.112\,\mathrm{m^2}$
Inrun lift area (C_lA_{Inrun})	$0.028\,\mathrm{m^2}$
Take-off drag area ($C_dA_{Take-off}$)	$0.340\,\mathrm{m^2}$
Take-off lift area ($C_lA_{Take-off}$)	$0.200\,\mathrm{m^2}$
Take-off speed	$2.80\,\mathrm{m\,s^{-1}}$
Air temperature	$0.0\,^{\circ}\mathrm{C}$

C_dA_{Inrun} and C_lA_{Inrun} were used from the start of the inrun to $6\,\mathrm{m}$ before the take-off, from this point C_dA and C_lA were interpolated linearly to $C_dA_{Take-off}$ and $C_lA_{Take-off}$.

Figure S2 shows the aerodynamic polar function for all five suits used for the numerical simulations. Figure was based on the polar function of Suit 1 and adjusted accordingly to Table S2.

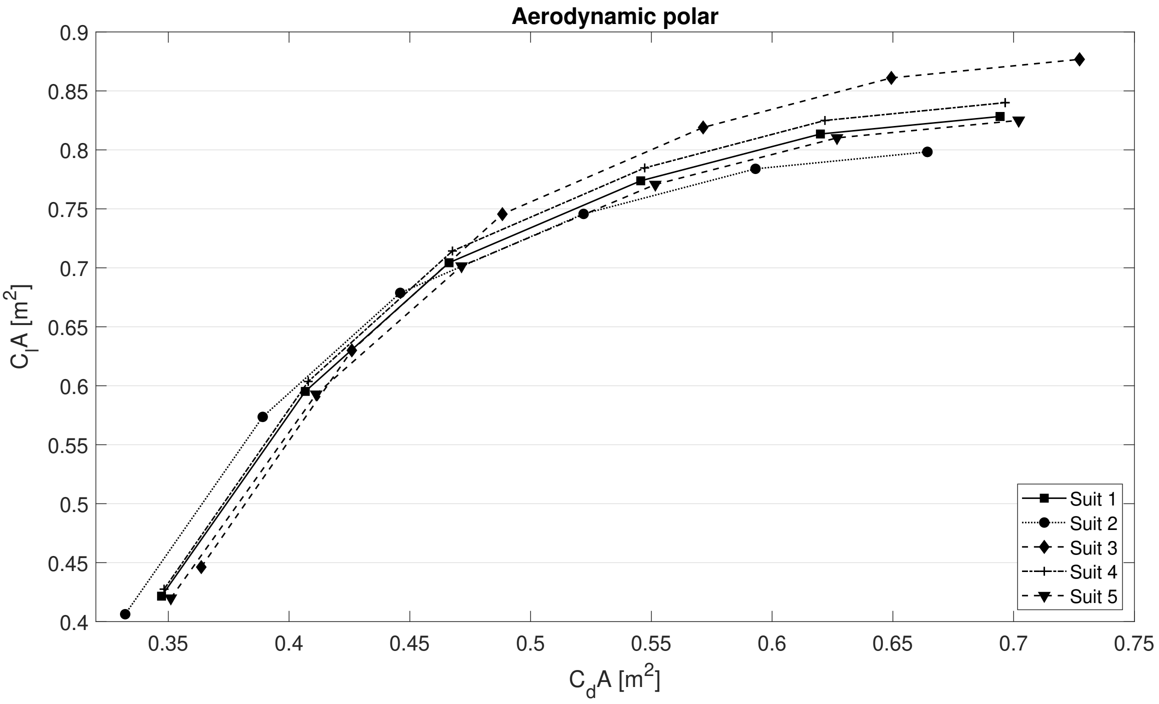

Figure S2. Aerodynamic polar function for all five suits, based on the wind tunnel results. Drag area (C_dA) on the horizontal axis, lift area (C_lA) on the vertical axis.

For the final part of this investigation, Suit 1, Suit 2 and Suit 3 were simulated to a jump length of $130\,\mathrm{m}$ by changing the inrun length. Figure S3 shows the trajectory in terms of meter over ground and the change in horizontal and vertical velocity, relative to Suit 1.

a.

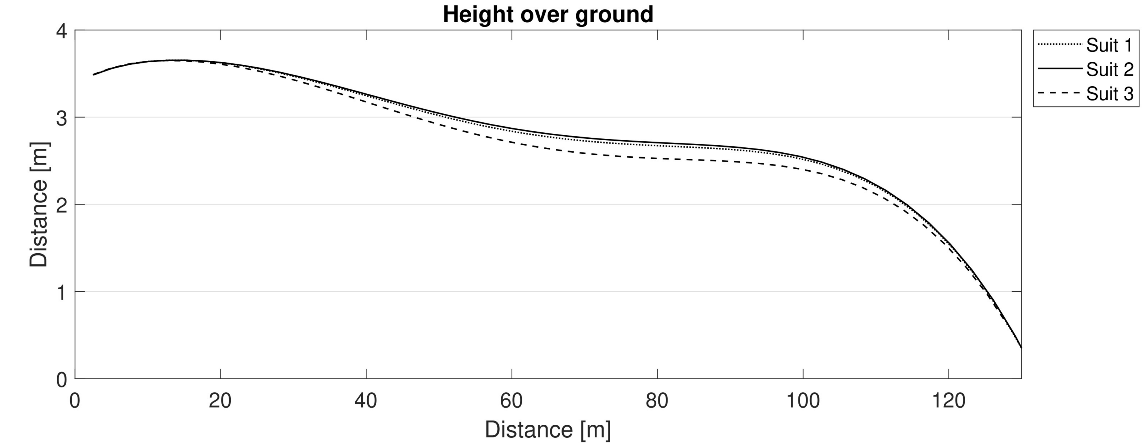

b.

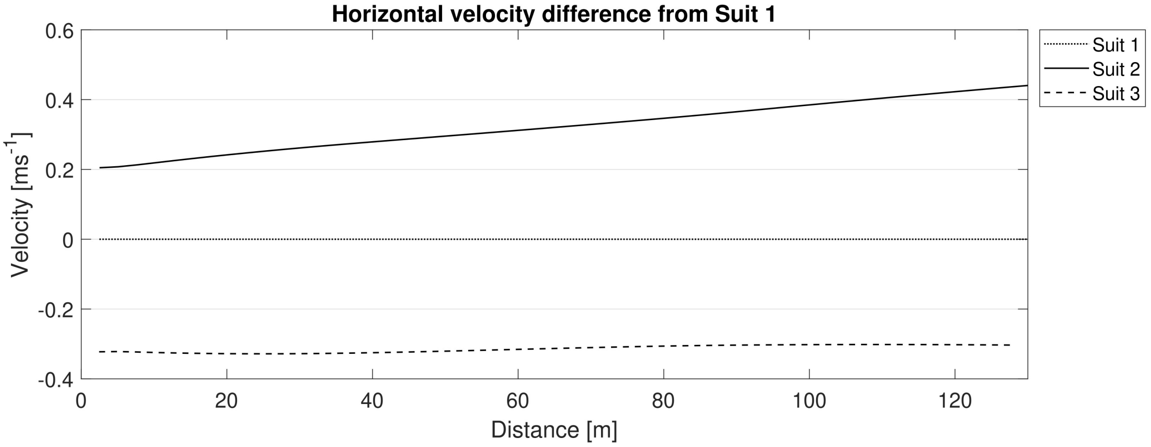

c.

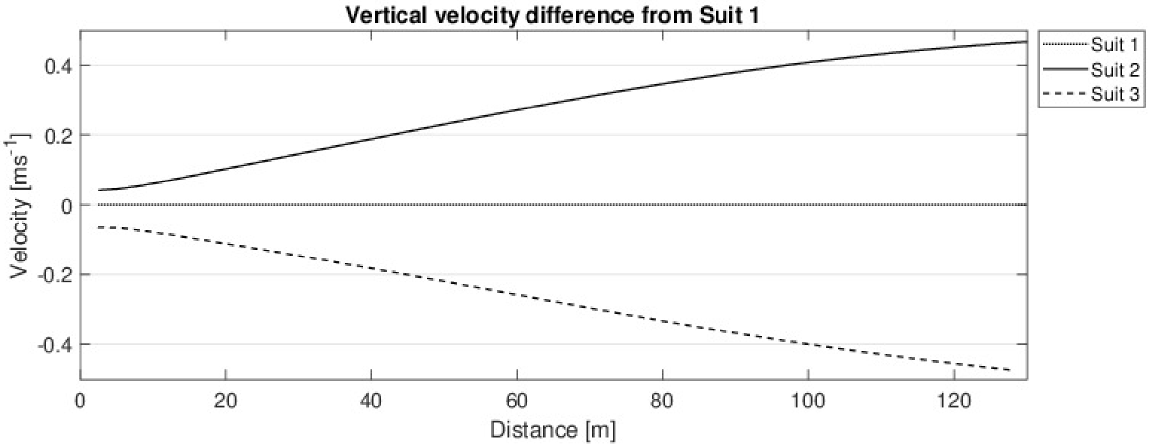

Figure S3. Comparison of suit 1, 2 and 3, i.e., suit size. The flight trajectory in terms of meter over ground is shown in **a**, horizontal and vertical velocity difference from Suit 1 in **a** and **b**, respectively.

Table S4 display information on all simulated jumps.

Table S4. Data on altitude, air density, wind conditions, start gate, inrun speed and jump length of all simulated jumps.

Suit	Altitude [m]	Air density [$\mathrm{kg\,m^{-3}}$]	Wind [$\mathrm{m\,s^{-1}}$]	Start gate [#]	Inrun Speed [$\mathrm{m\,s^{-1}}$]	Jump length [m]
Suit 1	500	1.22	0.0	14	24.92	130.0
Suit 2						125.3
Suit 3						136.5
Suit 4						131.9
Suit 5						126.7
Suit 1	500	1.22	-1.5	21	25.53	130.0
Suit 2						125.6
Suit 3						136.1
Suit 1	500	1.22	1.5	7	23.44	130.0
Suit 2						125.0
Suit 3						136.8
Suit 1	0	1.29	0.0	11	24.56	130.0
Suit 2						125.2
Suit 3						136.6
Suit 1	1000	1.15	0.0	16.5	25.19	130.0
Suit 2						125.4
Suit 3						136.3
